# Reconsidering the Meaning of Curing Primary Breast Cancer as a Systemic Disease

**DOI:** 10.3389/fonc.2021.639420

**Published:** 2021-03-18

**Authors:** Ryungsa Kim, Takanori Kin

**Affiliations:** ^1^Department of Breast Surgery, Hiroshima Mark Clinic, Hiroshima, Japan; ^2^Department of Breast Surgery, Hiroshima City Hospital, Hiroshima, Japan

**Keywords:** breast cancer, adjuvant therapy, neoadjuvant therapy, residual tumor cells, antitumor immunity

Over the past 50 years, advances in our understanding of the biology of breast cancer have revealed that it is not a single disease, but a heterogeneous group of cancers characterized by high genomic instability, as represented by somatic gene mutations, copy number alterations, and chromosomal structure rearrangement at the molecular level (1). Histologically, breast cancer is classified as invasive or non-invasive based on the presence or absence of myoepithelium, and further classified according to the presence or absence of hormone receptors (HRs; estrogen and progesterone receptors) and human epidermal growth factor receptor 2 (HER-2). Recent advances have enabled the identification of tumor subtypes based on gene expression profiles, leading to the classification of breast cancer cases as luminal (HR-positive/HER-2–negative), luminal–HER-2 (HR-positive/HER-2–positive), HER-2, and triple negative (TN) (2). Such subtyping has enabled the application of more definitive therapies targeting HR and HER-2, as well as prognosis prediction. In addition, pathological staging according to tumor biological characteristics could more accurately reflect the prognoses of patients in clinical practice than can classical pathological staging according to anatomical characteristics (3). Based on these backgrounds, the long-term outcomes of patients with breast cancer who have received adjuvant treatments, such as chemotherapy and endocrine therapy, have improved dramatically with the development of anticancer and endocrine agents (4–6). This approach derives from the concept of breast cancer as a systemic disease, first proposed by Bernard Fisher in the 1970s as an alternative to the 19th-century Halstedian theory of breast cancer progression (7). Dr. Fisher substantiated this concept in several preclinical and clinical studies in the 1970s, demonstrating (i) that primary tumor cells reach the lymph nodes through the lymphatics, where they are eradicated by antitumor immunity or metastasized, depending on the balance of host defense immunity and tumor cells; and (ii) that primary tumors release circulating tumor cells into the blood and transverse organs, some of which undergo endothelial attachment and promote tumor cell growth, resulting in tumor cell dissemination. From the perspective of surgical treatment, this concept was substantiated in two randomized controlled trials [National Surgical Adjuvant Breast and Bowel Project (NSABP) B-04 and B-06] (8, 9), which showed that total mastectomy is equivalent to radical mastectomy and that lumpectomy with radiation is equivalent to total mastectomy in terms of overall survival (OS). Thus, these trials demonstrated that extensive local surgical treatment involving the pectoral muscles, axillary lymph nodes, and entire breast did not improve the OS of patients with breast cancer. However, they represent early research; the concept of breast cancer as a systemic disease has been substantiated further by retrospective analyses showing that the addition of single-agent or combination adjuvant chemotherapy (AC) after surgical treatment yields positive results, suggesting that breast cancer can develop with distant metastasis in the early stage (10, 11). Many subsequent prospective randomized controlled trials have examined the survival benefits conferred by postoperative adjuvant therapies (i.e., endocrine therapy, anticancer agents, molecular targeted agents) relative to surgery alone in patients with breast cancer. With the advent of new agents, this survival benefit has grown significantly, with reduction of the breast cancer recurrence rate. Several obstacles, however, continue to block the achievement of a curative effect of adjuvant therapy, as evidenced by breast cancer recurrence with distant metastasis after such therapy. Several issues must be considered in the effort to achieve a breast cancer cure, taken as 0% distant recurrence.

First, the population in which adjuvant therapy confers a survival benefit has not been identified clearly. For example, the Milan trial, the first prospective randomized trial examining AC, demonstrated a survival benefit of the cyclophosphamide/methotrexate/5-fluorouracil regimen relative to surgery alone in patients with operable breast cancer (12), but a more specific population was not identified. There may be a population in which surgical treatment alone, without chemotherapy, is sufficient. Based on the results of that initial trial, anthracycline, taxanes, and dose-dense regimens were integrated into AC and tested against standard regimens, although endocrine therapy continued to be administered to patients with HR-positive breast cancer (13–15). The discovery of intrinsic tumor subtypes classified by multigene assays in the 2000s has clarified the indications for AC. In particular, recurrence scores obtained using the Oncotype Dx 21-gene assay guide the selection of patients with luminal (HR-positive/HER-2–negative) breast cancer who will benefit from AC (16–18). This approach represents a dramatic advance, enabling the avoidance of unnecessary AC and the selection of a population in which a survival benefit is expected, but the indications for AC are distinct from the efficacy of treatments in reducing distant metastatic recurrence and thereby improving survival. Currently, we cannot distinguish responders from non-responders prior to AC administration, and this treatment clearly will not improve survival in non-responders.

Second, the efficacy of AC and/or endocrine therapy is assessed according to the appearance of recurrence with distant metastasis (e.g., to the bone, distant lymph nodes, lung, liver, or brain). There is no way to predict or estimate this efficacy before, during, or after treatment. Thus, recurrence with distant metastasis is observed, although infrequently, even after the administration of standard treatment according to the tumor subtype and pathological stage. Several studies have demonstrated that the level of circulating tumor DNA, detected by liquid biopsy, is related to efficacy and prognosis for metastatic breast cancer treated with anticancer agents and early-stage breast cancer treated with neoadjuvant chemotherapy (NAC) (19–21), but no such assessment method is available for adjuvant therapy. As breast cancer is a systemic disease, residual or circulating tumor cells may be present after surgical treatment. Immune responses elicited by certain host immune defenses or adjuvant therapy can eradicate these cells, regardless of whether they have seeded in a metastatic niche, thereby curing the patient of breast cancer. Unfortunately, the immune responses involved in this eradication remain unidentified. Residual tumor cells may be killed by the cytotoxic effects of anticancer agents or the cytostatic effect of endocrine therapy, or these treatments may induce dormancy via the anti-estrogen effects of selective estrogen receptor modifiers or growth factor deprivation by aromatase inhibitors. In addition, remaining tumor cells that are resistant to anticancer agents or endocrine therapy may survive and proliferate, evolving into distant metastasis several years after adjuvant therapy. There is no way to monitor the residual cell–eradicating effects of adjuvant therapy to ensure that patients are cured of breast cancer.

Third, NAC has caused a paradigm shift due to the ability to predict efficacy and prognosis for patients with breast cancer treated with anticancer agents before surgery (22). The achievement of pathological complete response (pCR) after NAC predicts a good prognosis for HER-2 and TN breast cancers, but not for the luminal type because endocrine therapy affects the long-term outcomes of patients with HR-positive breast cancer (23, 24). Furthermore, patients who achieve pCR after NAC rarely relapse, depending on the definition of post-NAC pCR; patients in whom axillary lymph-node metastasis (Ax+) and the primary tumor have disappeared have a higher survival rate than do those without Ax+ disappearance (25). However, a meta-analysis demonstrated that increased pCR rates do not correlate simply with improved long-term outcomes (26), likely due to tumor heterogeneity, differences in the effects of adjuvant treatments (e.g., endocrine therapy and HER-2 targeted therapy), and the presence of residual tumor cells at the molecular level. Such cells can be detected by digital polymerase chain reaction (27, 28), but whether they can cause distant metastasis in patients who have achieved pCR remains unclear. Other potential benefits of NAC include its activation of the immune response, which contributes to primary tumor shrinkage and the eradication of metastatic tumor cells in axillary lymph nodes. The presence of tumor-infiltrating lymphocytes (TILs) before NAC is important for the achievement of a better therapeutic effect in patients with breast cancer; high TIL levels are associated with high post-NAC pCR rates, regardless of tumor subtype. However, the failure to achieve pCR, with the presence of residual tumor, does not necessarily confer a poorer prognosis than pCR for luminal breast cancer, in contrast to HER-2 and TN breast cancers; indeed, non-pCR has been associated with better survival than pCR for this tumor subtype (29), suggesting that TILs play a distinct functional role in eradicating luminal tumor cells after endocrine therapy. In contrast, tumor cell eradication in patients receiving NAC is achieved via immune activation by TILs, including T lymphocytes and natural killer (NK) cells. In patients with HER-2–positive breast cancer treated with trastuzumab, Fcγ receptor–mediated activation of NK cells is associated with marked improvement of the therapeutic effect (30). Moreover, the release of tumor-associated antigens from dead tumor cells, triggered by the cytotoxic effects of anticancer agents, may provoke antitumor immunity via cytotoxic T lymphocytes through priming by dendritic cells. Thus, NAC conceivably induces antitumor immunity, which may be involved in primary tumor shrinkage and the eradication of metastatic tumor cells.

Comparison of the efficacy of AC and NAC raises the important question of how immune activation for the complete eradication of tumor cells differs between these treatments. If the administration of anticancer agents to tumor cells before chemotherapy induces antitumor immunity and cures breast cancer, then NAC should be superior to postoperative AC. Unfortunately, two prospective randomized controlled trials (NSABP B-18 and B-27) comparing NAC, postoperative AC, and NAC with postoperative AC failed to show that NAC confers a survival benefit (31–33), although combination treatment with anthracyclines and taxanes doubled the pCR rate in the B-27 trial. However, the survival rate was significantly better in patients who achieved pCR after NAC than in those who did not in both studies. Analysis of a subset of data from the B-18 trial showed that disease-free survival (DFS) tended to be superior in patients with HR-negative breast cancer aged < 50 years who received NAC than in those who received AC (33). DFS also tended to be better in patients who received preoperative doxorubicin/cyclophosphamide followed by docetaxel, but this difference was not significant (32, 33). The addition of docetaxel after preoperative doxorubicin/cyclophosphamide therapy was found to significantly improve relapse-free survival (33). A meta-analysis showed that local recurrence rates were higher in patients with breast cancer treated with NAC than in those treated with AC, but that the breast cancer mortality rate did not differ between these groups (34). These results indicate that NAC may reduce breast cancer recurrence, but does not confer a survival benefit relative to postoperative AC. Nevertheless, NAC appears to activate immune responses in specific populations of patients with luminal, HER-2–positive, and TN breast cancers. The key benefit of NAC is the ability to predict long-term outcomes according to treatment responsiveness, and the targeting of residual tumor cells is a sound rationale for the administration of AC after NAC to improve survival (35, 36).

How should we define the curing of breast cancer after adjuvant therapy? Follow-up to monitor the presence/absence of residual tumor cells is currently performed for up to 10 years after surgical treatment. Breast cancer is considered to be cured in the absence of distant recurrence during this 10-year period, although a small percentage of recurrence is observed after 10 years. The hypothetical time course for the curing of breast cancer or development of recurrence with distant metastasis after adjuvant therapy is summarized in Figure 1. Tumor volume at diagnosis may be reduced macroscopically to the maximum degree by surgical treatment, and putative residual tumor cells can be eradicated by postoperative radiotherapy and AC and/or endocrine therapy (Pattern 1). Although most tumor cells may eventually be eradicated by antitumor immunity or endocrine therapy, a minimum number of resistant cells (minimal residual disease) may persist (Pattern 2). Persistent tumor cells may be regulated for years by antitumor immunity and endocrine therapy, may cause recurrence with distant metastasis due to additional genetic mutation with clonal activity, or may disappear due to host defense immunity (Pattern 3). According to the immunoediting aspect of cancer (37), persistent residual tumor cells are in equilibrium or in a dormant state and do not progress to distant metastasis. Eventually, however, cells that are not eliminated and escape the effects of antitumor immunity can cause distant metastasis. Thus, the balance between residual tumor cells and host defense immunity may play an important role in the eradication of these residual cells after AC or during endocrine therapy.

**Figure 1:**
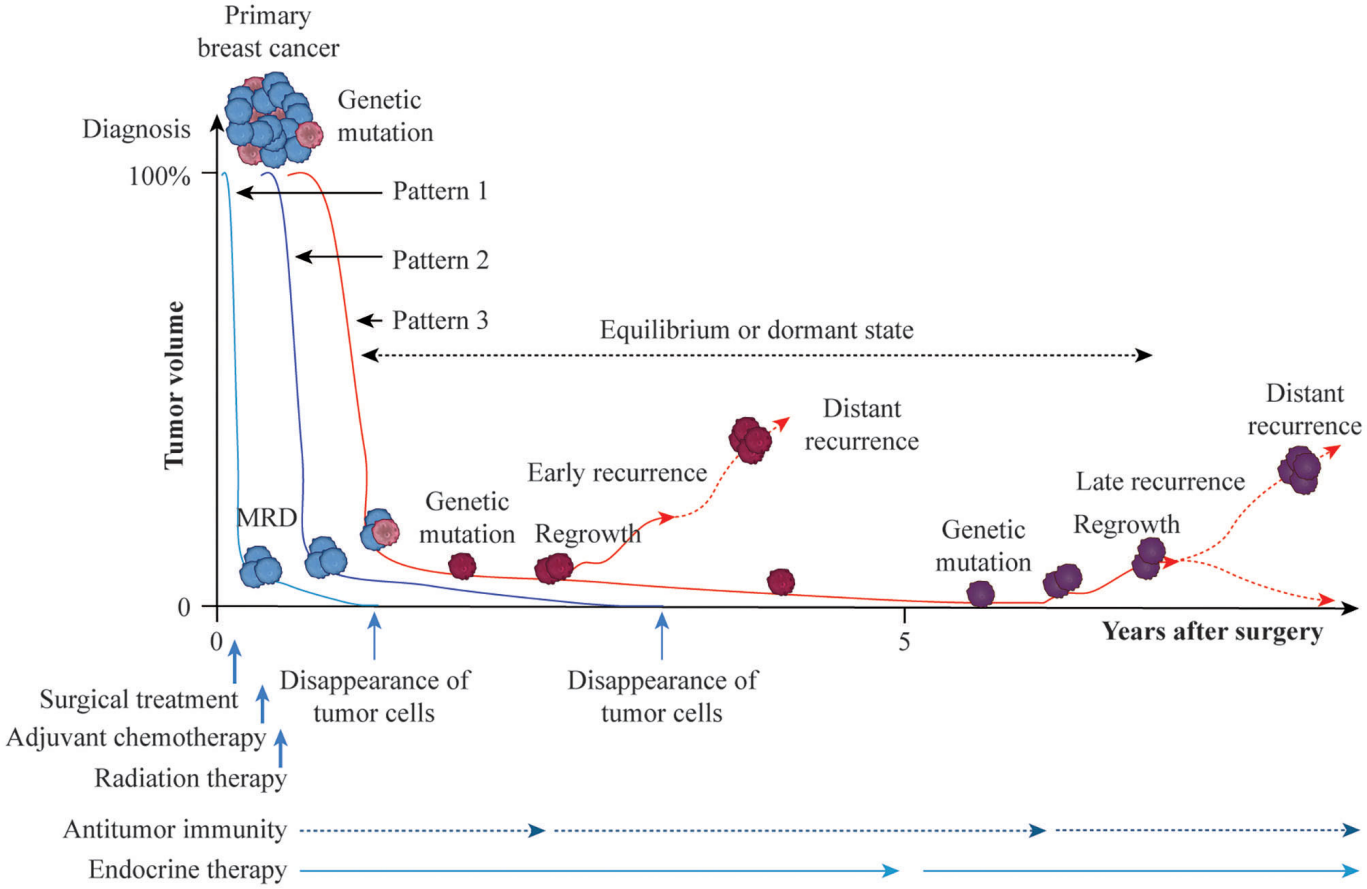
Possible time courses leading to breast cancer cure or recurrence with distant metastasis after adjuvant therapy. Breast cancer is a heterogeneous population with genetic mutations. Surgical treatment reduces the primary tumor volume at diagnosis, arbitrarily set to 100%, and axillary lymph-node metastasis to near 0% macroscopically. Then, adjuvant and radiation treatments are administered to eradicate putative residual tumor cells at the microscopic or molecular level (MRD, minimal residual disease). If adjuvant therapy is successful against residual tumor cells without genetic mutations, breast cancer is cured by the cytotoxic effects of anticancer agents, cytostatic effect of endocrine therapy, or induction of antitumor immunity (Pattern 1). Residual tumor cells without genetic mutations may persist for years in equilibrium or in a dormant state and then eventually disappear, leading to a cure (Pattern 2). Persistent residual tumor cells with genetic mutations are resistant to adjuvant therapy and may proliferate and cause distant metastasis as early recurrence with additional genetic mutations or remain in equilibrium or in a dormant state for several years, eventually disappearing, resulting in prolonged recurrence-free survival due to the effects of endocrine therapy or antitumor immunity. Otherwise, regrown tumor cells may cause distant metastasis as late recurrence with further genetic mutations (Patten 3).

Since the initiation of breast cancer control by surgery alone in a prospective randomized controlled trial conducted 50 years ago, such trials have led to dramatic advances in AC and endocrine therapy, with the de-escalation of breast cancer surgery and escalation of adjuvant treatment providing significant survival benefits for patients with all types of breast cancer. Recent advances in molecular targeted therapy and genomic analysis may make the development of personalized and tailored adjuvant therapies more accessible, but it is doubtful whether such strategies can achieve 0% distant recurrence of breast cancer. Prospective randomized controlled trials cannot detect small survival benefits in specific populations due to tumor heterogeneity and differences in treatment response. Strategies for the identification of residual tumor cells and targeted therapy to eradicate them, and, more importantly, the development of effective treatment for tumors resistant to adjuvant therapy (as determined by their genomic and molecular profiles) and the elucidation of the host immune defense mechanisms involved in residual tumor cell eradication are needed to achieve a complete cure for breast cancer after surgical treatment.

Genetic mutation and environmental exposure (e.g., lifestyle factors) are two important factors in breast cancer development, and the complex interaction between them is believed to be responsible for the existence of the disease. Lifestyle factors such as alcohol consumption, smoking, obesity, unhealthy diet, and low physical activity levels have been implicated in the risk not only of breast cancer development, but also of tumor progression and recurrence. A healthy lifestyle may help to prevent breast cancer or improve its prognosis, thereby contributing to the curing of primary breast cancer (38). The interaction of the gut microbiome with host defense immunity has also been associated with breast cancer. The human breast has a diverse microbial community distinct from those of other body sites. The diversity of breast bacteria is comparable to that observed in other microbial compartments, such as the gut, and this bacteria is likely translocated from the gut microbiome, potentially via skin/nipple-areolar orifices or blood (39). A recent whole-genome analysis led to the detection of a distinct pattern of microbial signatures specific to the TN and triple-positive breast cancer subtypes, with similar microbial signatures detected in the ER-positive and HER-2–positive breast cancers (40). The observation of microbial dysbiosis among normal breast, tumor, and tumor-adjacent tissues suggests that bacteria or their components in the microenvironment affect the local immune defense (41, 42). However, how dysbiosis in breast tissue contributes to breast cancer development in host immune defense remains unclear. Commensal dysbiosis may promote tissue inflammation and tumor cell dissemination through crosstalk between the tumor and tissue environments, and dysbiosis of the gut microbiome may play a role in breast cancer progression similar to that of estrogen in the progression of HR-positive breast cancer (43). A better understanding of the role of host–microbiome interactions in the gut and breast in host defense immunity may lead to the development of new therapeutic strategies and improved prognosis of primary breast cancer.

Breast cancer is characterized by tumor heterogeneity and resistance to anticancer agents and endocrine therapy, and thus to curative treatment. Two models of tumor heterogeneity have been proposed (44): a clonal evolution model in which random genetic mutations and clonal selection cause cellular heterogeneity in breast tumors (45) and a stem cell model, which consists of cell diversity and hierarchical organization in tumors generated by breast cancer stem cells (BCSCs) (46). Random genetic mutations followed by clonal evolution can result in the upgrading of malignant tumors with the potential for metastasis during tumor progression. During metastasis, various types of protease are required to invade the extracellular matrix. Among them, lysosome plays an important role in the invasion and metastasis of breast cancer cells, which are mediated by the pathways, regulation, and secretion of cathepsins and other proteases (47). During tumor progression, cathepsins are secreted into the extracellular space to facilitate direct cleavage of the extracellular matrix and membrane, the migration of tumor cells from the primary lesion, the activation of other proteases such as matrix metalloproteinases and urokinase plasminogen activator, and cleavage of the cell adhesion protein E-cadherin to promote tumor migration and invasion (48). During breast cancer progression, tumor cells acquire resistance to acid-induced toxicity and extracellular matrix degradation through the release of cathepsin B into the tumor microenvironment, providing for acid-mediated tumor invasion with altered glucose metabolism (49). The lysosomes of cancer cells at the invasive edges of tumors were found to be redistributed from the perinuclear region to the peripheral region, and lysosomal trafficking in these cells was found to be altered (50). Lysosome is also involved in the lysosomal cell death pathway, a caspase-independent and Bcl-2–insensitive pathway in apoptosis-resistant cells that causes autophagic cell death (51). Autophagy is generally considered to consist of the biphasic biological phenomenon of cell survival and cell death, depending on the cell type, physical circumstances, and stimulus (52). The serine threonine kinase Akt plays an important role in autophagy, and the lysosomal accumulation of Akt is an essential step in autophagy induction (53). Recent preclinical studies have indicated that the inhibition of autophagy does not impair the functions of T cells against breast cancer cells, including those that have received chemotherapy (54). Other studies have suggested that autophagy protects tumor immunity by promoting the degradation of cytolytic granules of T and NK cells (55, 56). Lysosome inhibition may be a breakthrough strategy for the improvement of therapeutic efficacy and overcoming of resistance to anticancer agents. Further studies are needed to inform the development of lysosome-targeting anticancer therapies and the overcoming of breast cancer drug resistance via the use of the lysosomal cell death pathway in patients with breast cancer.

BCSCs are potential candidates for drug resistance; they have been detected with the most commonly used molecular markers for CD44-positive/CD24-negative/low cells and aldehyde dehydrogenase 1–positive cells, and are associated with poor prognosis (57, 58). These cells have marked self-renewal and differentiation abilities, and constitute phenotypically diverse populations that can reconstitute the heterogeneity of primary tumors of various subtypes. Further studies are needed to better characterize BCSCs and to develop BCSC-targeted therapies, including those based on microRNA. Given that early detection is the key to curing breast cancer, and that primary treatment (surgical treatment, radiation therapy and neoadjuvant or adjuvant therapy) provides the only opportunity for a cure, the identification of a population of patients who will benefit from adjuvant therapy, and the development of targeted therapies for patients who cannot overcome such resistance, can lead to the achievement of 0% distant recurrence of breast cancer. Further definitive research is required to reach this goal.

The development of innovative therapies targeting new proteins and pathways associated with the growth of breast cancers lacking the HER-2 protein could improve breast cancer treatment. Immunotherapy [e.g., that based on immune checkpoint (IC) inhibitors], may be a promising strategy leading to the curing of breast cancer, especially the TN subtype. Antibodies against programmed cell death protein 1 (PD-1) and programmed death ligand 1 (PD-L1) are used to stimulate patients' immune responses, and thus the recognition and destruction of cancer cells (59). These therapeutic effects can be enhanced by combined treatment with anticancer agents. Currently, combination therapies with IC inhibitors of PD-1 and PD-L1 and anticancer agents in neoadjuvant and adjuvant settings are being assessed, ae well as metastatic breast cancer (60). Previous neoadjuvant therapy studies yielded conflicting results (61–63); we await data from ongoing clinical trials indicating which IC inhibitors are effective and suitable for use in combination with anticancer agents. The identification and targeting of actionable mutations or alterations in tumors will enable the design of personalized approaches to breast cancer treatment, individualized for patients based on the goal of breast cancer cell eradication or control. Further extensive research in multiple fields, including the surgical, medical, and radiation oncology disciplines, in conjunction with basic research is required.

## Author Contributions

All authors were responsible for the conceptualization of this perspective, writing of the manuscript, approval of the final draft, and also responsible for the accuracy, integrity, and accountability of this work.

## Conflict of Interest

The authors declare that the research was conducted in the absence of any commercial or financial relationships that could be construed as a potential conflict of interest.
